# Integration of traditional medicine into the mental healthcare system in Tshwane, South Africa

**DOI:** 10.4102/safp.v65i1.5636

**Published:** 2023-05-19

**Authors:** Vusi F.J. Masemola, Ellen M. Thobakgale, Indiran Govender

**Affiliations:** 1 Department of Nursing, Faculty of Health Care Sciences, Sefako Makgatho Health Sciences University, Pretoria, South Africa; 2 School of Health Care Sciences, Department of Nursing Sciences, Faculty of Health Sciences, University of Limpopo, Polokwane, South Africa; 3 Department Family Medicine and Primary Health Care, Faculty of Health Sciences, Sefako Makgatho Health Sciences University, Pretoria, South Africa

**Keywords:** attitude, integration, mental healthcare providers, psychiatric hospital, traditional health medicine

## Abstract

**Background:**

Mental healthcare providers have a negative attitude towards the integration of traditional health medicine (THM) into the mental health system. The attitude is based on their lack of trust in traditional practices, which are not supported by substantial evidence-based medical research. The study was conducted to determine mental healthcare providers views on the integration of traditional health medicine into the mental healthcare system.

**Methods:**

The study was conducted at a psychiatric hospital in the Tshwane district, Gauteng province, South Africa. A cross-sectional, descriptive research design was conducted on 85 respondents who consented to participate. Data were collected using a survey questionnaire from 23 psychiatrists and 62 psychiatric nurses. Data were analysed using descriptive statistics and presented in the form of graphs, frequencies and percentages.

**Results:**

Mental healthcare providers displayed a negative attitude towards integrating THM in psychiatric hospitals. The results showed no significant difference between psychiatrists and psychiatric nurses in their preference for modern mental healthcare practices (*p* = 0.25).

**Conclusion:**

There is still doubt among the mental healthcare providers on whether to support integration of the THM into mental health system or not. The doubt is based on the two-health system isolated from each other especially in South Africa.

**Contribution:**

This study contributed by showing the need and importance of understanding a patients’ cultural background, which supports the integration of a traditional health system into the mental healthcare system, which in turn will lead to the appropriate management of mental illnesses.

## Introduction

Attitudes among mental healthcare providers towards integrating traditional health medicine (THM) in psychiatric hospitals were reported to be unsatisfactory.^1^ Mental healthcare providers’ attitudes towards THM are based on their lack of trust in traditional practice, attributed to the lack of strong research evidence.^2^ Mental healthcare providers’ understanding of mental healthcare users’ (MHCU) cultural backgrounds is essential to manage and advise users accordingly.^3^ In the South African context, according to the Traditional Health Practitioners Bill (25 of 2004),^4^ mental healthcare providers are continuously encouraged to have a deeper understanding of traditional medicine, which improves the mental health of individuals because many patients use traditional medicine. In KwaZulu-Natal, eThekwini, traditional health practitioners (THPs) have collaborated with the Department of Health on cross-education about preventing illnesses, voluntary counselling and referrals.^5^ The South African government supports the integration of THM into the mental healthcare system.^6^

The Ghanaian government also supports similar integration. However, there is still resistance from the biomedical health system to recognise THM.^7^ The herbal medicine industry is expanding globally, and these herbs are advertised on the radio and in newspapers.^8^ Some THMs are accessible to everyone, and their use continues in high numbers among some mental healthcare providers.^9^ Although little research has been conducted on integrating traditional practice into the mental healthcare system, studies show a willingness to learn about THM.^10^ Thus, this study aimed to determine the mental healthcare providers’ attitudes towards integrating THM into the biomedical mental healthcare system in Tshwane district, Gauteng province, South Africa.

## Methodology

This was a quantitative descriptive research design. The mental healthcare centre consists of 48 psychiatrists and 248 professional nurses. Other staff categories were excluded including dieticians, physiotherapists, occupational therapists, psychologists and social workers. The sample consisted of 85 mental healthcare providers who agreed to participate from a psychiatric hospital in Tshwane district municipality in Gauteng province. Of the 85 respondents, 23 respondents were psychiatrists and 62 were psychiatric nurses. Data were collected using a self-developed survey questionnaire distributed for completion by both day- and night-shift mental healthcare providers in all the psychiatric hospital wards. Distributing the questionnaire to both shifts ensured that mental healthcare providers from both shifts (day and night) were included. Questions were subjected to evaluation before finalisation; this was to ensure that they remained relevant to the study and were not ambiguous. Before the primary research study, the researcher distributed about 10 questionnaires in two wards at the hospital in Tshwane and they were completed by the respondents. Before completion of the questionnaire, they were asked to indicate if they understood all the questions and to provide comments on any questions that may need clarification. Those questionnaires were not included in the final analysis. Questionnaires were also sent to researchers to review and complete, and to check for relevance to the study objectives. Questions were formulated according to the study’s objectives and to test for internal consistency, Cronbach’s alpha was used. The results showed an internal consistency of greater than 0.78. Descriptive statistics were used to analyse the data, which were then presented in the form of graphs, frequencies and percentages calculated using the Epidemiologist Software (Epi Info™, Centers for Disease Control and Preventions, Atlanta, Georgia, United States). The scores were summarised and compared.

### Ethical considerations

Ethical clearance was obtained from the Sefako Makgatho Health Sciences University Research Ethics Committee (SMUREC) (reference: SMUREC/H/75/2017: PG) and the School of Health Care Sciences Research Committee (SHCSRC). Permission to conduct the study was requested and obtained from the Chief Executive Officer (CEO) of the psychiatric hospital, the nursing manager, and the clinical manager. Permission was also obtained from the mental healthcare providers after sharing the study’s purpose and objectives with the respondents. Anonymity and confidentiality were maintained, and respondents were instructed not to provide any identifying information.

## Results

### Demographic data of participants

The respondents’ average age was 38 years, with the youngest at 24 years and the eldest at 63 years. Results show that most of the respondents (31 of 85 or 36, 4%) were between the ages of 40 and 49 years.

### Mental healthcare providers’ attitude towards integrating traditional healthcare practices

Results in Table 1 show that 32% of the mental healthcare providers were concerned and desired to stop patients from using traditional medicines. The latter was followed by 29% of respondents (15% of psychiatric nurses and 4% of psychiatrists) who felt that it is the concern of the patients using traditional medicines and they have no reason to interfere. In comparison, 14% of respondents (14% of psychiatric nurses and no psychiatrists) felt concerned but tended to stay away from the patients using traditional medicines (Table 2). Results further showed that 11% of psychiatric nurses and 6% of psychiatrists felt concerned and wanted to give these patients the courage that they should continue with the hospital treatment and that they would improve (Table 2). The responses of psychiatric nurses and psychiatrists differed significantly regarding their feelings about patients using traditional medicines (*p* = 0.001).

**TABLE 1:** The response of mental healthcare providers on attitude.

Response	*N*	%	Yes		No		Not sure	
			*n*	%	*n*	%	*n*	%
**Do you approve of traditional healthcare practice?**								
Psychiatric nurses	62	100	28	45	22	35	12	20
Psychiatrists	23	100	9	41	9	41	4	18
All	85	100	37	44	31	37	16	19
**Do you consider traditional medicine to be safe?**								
Psychiatric nurses	62	100	12	20	19	30	31	50
Psychiatrists	23	100	4	18	9	41	9	41
All	85	100	16	19	28	33	40	48
**Do you feel a need to be given education by a traditional practitioner about traditional medicine?**								
Psychiatric nurses	62	100	44	71	13	21	52	8
Psychiatrists	23	100	17	77	5	23	0	-
All	85	100	61	73	18	21	5	6
**Do you support the cooperation of modern and traditional health practitioners and the integration of the two systems?**								
Psychiatric nurses	62	100	36	58	20	32	6	10
Psychiatrists	23	100	13	59	6	27	3	14
All	85	100	49	58	26	31	9	11
**Do you agree with traditional practitioners’ training on the mental healthcare system for the improvement of practice in the mental healthcare system? (support for integration)**								
Psychiatric nurses	62	100	43	69	6	10	13	21
Psychiatrists	23	100	18	82	3	14	1	4
All	85	100	61	72	9	11	14	17
**Do you agree with the government to support of traditional healthcare practitioners?**								
Psychiatric nurses	62	100	48	77	14	23	-	-
Psychiatrists	23	100	17	77	5	23	-	-
All	85	100	65	77	19	23	-	-
**Have you ever visited traditional practitioners for a consultation?**								
Psychiatric nurses	62	100	32	52	30	48	-	-
Psychiatrists	23	100	10	45	12	55	-	-
All	85	100	42	50	42	50	-	-

**TABLE 2:** Perceptions towards patients using traditional medicines.

Perceptions towards patients using traditional medicines	Psychiatric nurses (*N* = 62)		Psychiatrists (*N* = 23)		Total (*N* = 84)	
	*n*	%	*n*	%	*n*	%
I have no particular feeling.	13	8	4	1	-	9
It is their problem and I have no reason to interfere.	15	9	4	1	-	19
I feel concerned and desire to stop them.	20	12	12	3	-	15
I feel concerned and I am willing to give them the courage to continue taking the hospital treatment.	11	7	6	2	-	17
I feel concerned but tend to stay away from them and not intervene.	15	9	0	0	-	9

### The *p*-value while comparing opinions of professional nurses and psychiatrists = 0.001

In this study, 50% of mental healthcare providers have visited a THP for a consultation regarding a mental health condition (Table 3).

**TABLE 3:** Previous use of traditional medicine or traditional healers on a personal level.

Have you personally used traditional medicine or traditional healers previously?	Yes		No	
	*n*	%	*n*	%
Psychiatric nurses	32	38	30	36
Psychiatrists	10	12	12	14

**Total**	**42**	**50**	**42**	**50**

From Table 4 it is seen that only 19% of mental healthcare providers considered THM safe, although many had previously used THMs. The majority of respondents (73%) agreed that the THPs should be trained in the modern mental healthcare system.

**TABLE 4:** Views on the safety of traditional medicines and training for traditional practitioners about the modern (biomedical) mental healthcare system.

Views	Yes		No		Uncertain	
	*n*	%	*n*	%	*n*	%
**Are traditional medicines safe for mental healthcare use?**						
Psychiatric nurses	12	14	18	22	31	37
Psychiatrists	4	5	9	11	9	11
Total	16	19	27	33	40	48
**Should traditional health practitioners be trained in the modern mental healthcare system?**						
Psychiatric nurses	42	51	6	7	12	15
Psychiatrists	18	22	3	4	1	1
Total	60	73	9	11	13	16

**FIGURE 1:**
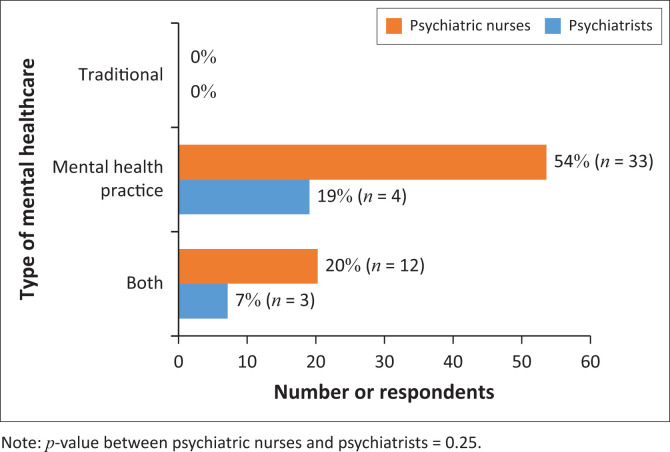
Healthcare provider preference.

The majority of mental healthcare providers (73%) preferred the mental healthcare practice (the majority being 54%) (Figure 1). The latter results were followed by 27% of respondents who indicated that they preferred both mental health practices. None of the participating mental healthcare providers (both psychiatric nurses and psychiatrists) preferred traditional medicine only. The results show no significant difference between psychiatrists’ and psychiatric nurses’ responses.

## Key findings

Traditional health practitioners are approved by 44% of the mental healthcare provider and only 37% do not approve followed by 19% who are not sure whether to approve the THPs or not. Previous research has shown that respondents who visited the THPs saw an improvement in mental illness and preferred that they be integrated into the mental healthcare system.^11^ Interestingly, this study shows that 50% of mental healthcare providers have personally visited a THP for consultation. A study in the United States shows that mental healthcare providers are likely to use THM because of their personal experience with Complementary Alternative Medicine. Mental healthcare providers working in biomedical institutions have reported that most of them in ambulatory care settings are exposed to THM.^12^ Thus, THP is a common practice, and mental healthcare providers must collaborate and learn more about it.

Results revealed that 48% of mental healthcare providers were uncertain about the safety of THM. Only 19% of mental healthcare providers considered THM to be safe. In the study, 31% of mental healthcare providers expressed a need to be educated about traditional medicine. The same sentiments were shared by the THPs in a study conducted in KwaZulu-Natal, South Africa. The majority of those respondents were more than willing to learn and support the integration of traditional medicine into modern medical healthcare practices.^13^

The study results have shown that respondents in the majority (49%) support integrating THP into the mental healthcare system. Only 5% of the respondents believe that it will not make any difference to the healthcare system. A study conducted by Mokgobi shows selective approval of the THP where most psychiatrists approved of only the herbalist to be integrated into modern mental healthcare practices.^14^

Some mental healthcare providers felt unconcerned and had no desire to advise MHCU using THMs. Some of these healthcare providers felt that they had no reason to stop patients from using traditional medicine as it is not their problem to interfere, and only a few felt concerned but preferred to stay away from these patients and not intervene. The majority of respondents (57%) agreed that the THPs should be trained in the modern mental healthcare system. The majority of respondents (73%) preferred the mental healthcare system. In contrast, none of the respondents preferred THP on its own as a system that can function independently. However, many nurses in primary healthcare systems have anecdotal information about THM use. This information from nurses is usually kept safe and such information is used as a reference when they want to advise or prescribe a particular traditional medicine.^13^ Thus, although many mental healthcare providers prefer the biomedical approach, they are aware of local THP. Health workers globally would like to see collaboration between THPs and biomedical practitioners but are concerned about side effects, and some may be unknown when combining the two types of treatments for mental health conditions.^14^ This study has shown that none of the mental healthcare providers believe that traditional health practices are sufficient on their own to manage mental healthcare diseases.

## Conclusion

It is concluded that the integration of traditional health practice into the mental health system is welcomed by the mental healthcare providers and it remains the responsibility of the government to ensure the effective implementation of the integrated healthcare system. Mental healthcare providers have demonstrated a willingness to learn about THM; moreover, some of them have personally used THM. The mental healthcare providers’ concern regarding THMs is the lack of clarity and plan to integrate THP into the mental healthcare system. The traditional health practice council’s scope and functions are more like the Health Professional Council of South Africa and the South African Nursing Council. The patients are not advised but rather discouraged from using THMs. Only a few mental healthcare providers in this study and previous studies are willing to discuss THP with patients and are aware of its safety profile.

It is recommended that the heads or managers of mental healthcare institutions encourage staff members to participate in research to benefit the institution and aid the government towards appropriate resource allocation and future planning. The introduction of THP in the mental healthcare providers’ curriculum at the universities remains a critical element of fast-tracking the integration of traditional health practice into the mental healthcare system.

Government must develop policies and structures that support the integration. Mental healthcare providers need to be made aware that they are obliged to impart health education and advise the patients based on their treatment of choice.

### Limitations of the study

The study was carried out in one institution only; therefore, the results are specific to that institution.
